# Prevalence of *mcr*-type genes among colistin-resistant *Enterobacteriaceae* collected in 2014-2016 as part of the INFORM global surveillance program

**DOI:** 10.1371/journal.pone.0195281

**Published:** 2018-04-02

**Authors:** Mark G. Wise, Mark A. Estabrook, Daniel F. Sahm, Gregory G. Stone, Krystyna M. Kazmierczak

**Affiliations:** 1 International Health Management Associates, Schaumburg, Illinois, United States of America; 2 AstraZeneca Pharmaceuticals, Waltham, Massachusetts, United States of America; Cornell University, UNITED STATES

## Abstract

A set of 908 clinically derived colistin-resistant *Enterobacteriaeae* isolates collected worldwide in 2014–2016 were screened for the presence of the plasmid-borne *mcr*-1, *mcr*-2, *mcr*-3, *mcr*-4 and *mcr*-5 genes. In total 3.2% (29/908) of the collection were positive for *mcr*, including 27 *Escherichia coli*, *1 Klebsiella pneumoniae* and 1 *Enterobacter cloacae*. Twenty-four isolates possessed genes from the *mcr*-1 family, including the original *mcr*-1 (n = 22), as well as *mcr*-1.2 (n = 1) and *mcr*-1.5 (n = 1), which each differ from *mcr*-1 by encoding single amino acid variations. Genes from the *mcr-*3 family were found in isolates from Thailand, including *mcr-*3.1 (n = 3) and *mcr-*3.2 (n = 1). An *E*. *coli* isolated from a patient with a urinary tract infection in Colombia contained the recently discovered *mcr*-5. The full colistin-resistant collection was tested against a panel of antimicrobial agents with ceftazidime-avibactam and tigecycline exhibiting the highest activity.

## Introduction

Use of colistin, which became clinically available in 1959, has historically played a minor role as an anti-infective therapy due to its nephrotoxicity, as well as the availability of alternative antimicrobial agents [1]. However, the recent proliferation of multi-drug resistant (MDR) Gram-negative pathogens in the clinical setting threatens the efficacy of antibiotics across all classes. To bolster the number of so called “last resort” antimicrobial agents, polymyxins such as colistin are once again being administered clinically due to their potential effectiveness against MDR infections [2]. Until 2015, all characterized colistin resistance mechanisms were chromosomally encoded and thus only limited vertical transmission of resistance was envisioned [3]. However, the discovery by Liu, et al. [4] of the plasmid-borne phosphoethanolamine transferase resistance determinant *mcr*-1 revealed a mechanism for horizontal spread. MCR-1 and MCR-2, a protein with 80.7% identity to MCR-1 [5], have now been reported in *Enterobacteriaceae* worldwide [6–8]. In 2017, three additional MCR protein variants have been described, MCR-3 [9], MCR-4 [10] and MCR-5 [11], all isolated from hosts with agricultural origins. To gain further insight into the global prevalence of *mcr* in enteric bacteria isolated from human clinical samples, colistin-resistant isolates from a large international surveillance study were examined for the presence of these genes.

## Material and methods

The INFORM (International Network for Optimal Resistance Monitoring) global surveillance program monitors antimicrobial resistance to a variety of pathogens isolated from intra-abdominal, urinary tract, skin/soft tissue, lower respiratory tract and, as of 2014, blood infections [12]. During 2014–2016, the program received a total of 44,407 isolates of *Enterobacteriaceae* including those collected by 87 medical center laboratories located in 18 countries in Europe (n = 21,461), 36 medical center laboratories in 9 countries in the Asia/Pacific region (n = 7,215), 24 medical center laboratories in 6 countries in Latin America (n = 7,180), 17 medical center laboratories in 5 countries in the Middle East/Africa region (n = 3,707) and 26 medical center laboratories in the United States (n = 4,844). All isolate species identifications were confirmed in the central laboratory by MALDI-TOF MS (Bruker Daltonics, Waltham, Massachusetts). Not including *Serratia* spp. and members of the tribe *Proteeae* (genera *Proteus*, *Providencia* and *Morganella*), which are intrinsically colistin non-susceptible, 934 isolates were found to be resistant to colistin by broth microdilution [13] at an MIC ≥ 4 μg/mL, which is the EUCAST resistance breakpoint for the *Enterobacteriaceae* [14]. Of these, 908 isolates were available to screen, as no isolates could be obtained from China in 2014–2016 or Hong Kong in 2015–2016 due to export restrictions. The species composition of the complete set included *Citrobacter freundii* (n = 6), *Citrobacter koseri* (n = 3), *Enterobacter aerogenes* (n = 18), *Enterobacter asburiae* (n = 143), *Enterobacter cancerogenus* (n = 1), *Enterobacter cloacae* (n = 165), *Enterobacter kobei* (n = 11), *Escherichia coli* (n = 64), *Hafnia alvei* (n = 1), *Klebsiella oxytoca* (n = 13), *Klebsiella pneumoniae* (n = 481) and *Klebsiella variicola* (n = 2).

The collection was investigated for the presence of the colistin-resistance conferring *mcr* genes by several PCRs. The initial reaction utilized a custom primer set designed to amplify a 143 bp region common to both *mcr*-1 and *mcr*-2 (MCR-Univ-F: 5’-CTGTGCCGTGTATGTTCAGC-3’ and MCR-Univ-R: 5’-CACGCCTTTTGAGTCYGAAT-3’). Primers that anneal to 16S rRNA gene (U341F, 5’-CCTACGGGRSGCAGCAG-3’; U519R 5’-GWATTACCGCGGCKGCTG-3’) were included in the reaction as an internal positive control for amplification. Subsequently, a multiplex PCR was employed with primers MCR3-F and MCR3-R [9], and MCR-4 FW and MCR-4 RV [10] to detect the *mcr-*3 and *mcr*-4 genes, respectively. This reaction also included the 16S rDNA internal positive control. Finally, the screening for *mcr*-5 utilized MCR5-intern_fw and MCR5-intern_rev primers [11], along with the internal 16SrDNA control. As external positive controls, synthetic DNA constructs were employed for each of the *mcr* genes (IDT Inc., Coralville, Iowa). All screen-positive results were confirmed by PCR amplification using custom-designed primers flanking the coding region and sequencing the gene in full (*mcr-*1, exgenMCR1-F, 5’-CCGYAATTATCCCACCGTTT- 3’ and exgenMCR1-F, 5’-CGCCATGACAAGAGCGATAC-3’; *mcr*-3, exgenMCR3-F, 5’-TCGTTAGAAAGTGATTGTTGGAC-3’ and exgenMCR3-R, 5’-CCTCTTTCTGATTTGCCCGT-3’; mcr-5, exgenMCR5-F, 5’-AACCGTTGAAAGAAGAGGACA-3’ and exgenMCR5-R, 5’-CCAATGAGCTCGTGATCCCC-3’). Sequence variants were assigned based upon comparison to sequences deposited in the NCBI databases. *mcr*-positive *E*. *coli* underwent multilocus sequence typing based on the partial sequences of *adk*, *fumC*, *gyrB*, *icd*, *mdh*, *purA*, and *recA* (https://enterobase.warwick.ac.uk/species/index/ecoli).

## Results and discussion

In total, *mcr* was detected in 29 isolates (3.2%), and included 27 *E*. *coli*, 1 *K*. *pneumoniae* and 1 *E*. *cloacae* collected in 15 countries (Malaysia, 5; Thailand, 5; Spain, 3; Argentina, 2; Italy, 2; Colombia, 2; Germany, 2; Brazil, Hong Kong, Poland, Portugal, Russia, South Africa, Taiwan, and Venezuela, 1 each) as part of INFORM in 2014 (n = 14), 2015 (n = 11) and 2016 (n = 4) (Table 1). Twenty-two isolates harbored the original *mcr-*1 gene, one isolate carried the gene for the single amino acid variant (Q3L) MCR-1.2 [15], and one isolate carried *mcr*-1.5, that codes for another single amino acid variant, (H452Y). Four *E*. *coli* isolates, all originating from Thailand, were found to possess *mcr-3*, with three harboring the original *mcr-*3.1 [9] and one possessing the gene coding for the single amino acid variant, MCR-3.2 (T488I). An *E*. *coli* strain from Colombia was shown to carry the recently discovered *mcr*-5 gene [11]. No *mcr-2* or *mcr-4* genes were identified.

**Table 1:** *mcr* positive *Enterobacteriaceae* collected as part of the INFORM global surveillance program during 2014–2016.

Year	Country	Organism	Clinical Sample	MIC (μg/mL)^a^					MLST	*mcr* gene product	β-Lactamase content^b^
				CST	CAZ-AVI	CAZ	MEM	TGC			
2014	Colombia	*Escherichia coli*	Urine	4	0.25	32	0.06	0.25	ST641	MCR-5	CMY-2
2014	Germany	*Escherichia coli*	GI tract: appendix	>4	0.06	0.25	0.03	0.12	ST46	MCR-1	NC^c^
2014	Hong Kong	*Escherichia coli*	Blood	4	0.06	0.12	0.03	0.25	ST10	MCR-1	NC
2014	Italy	*Escherichia coli*	Wound	4	0.12	0.25	0.015	0.25	ST744	MCR-1	NC
2014	Italy	*Escherichia coli*	Blood	4	0.12	0.25	0.015	0.25	ST453	MCR-1.2	NC
2014	Malaysia	*Escherichia coli*	Abscess	4	0.12	16	0.03	1	ST10	MCR-1	TEM-OSBL^d^; CTX-M-15
2014	Malaysia	*Escherichia coli*	Gangrene	4	0.03	16	0.03	0.5	ST162	MCR-1	TEM-OSBL; CMY-2
2014	Portugal	*Enterobacter cloacae*	Wound	>4	0.25	1	0.06	1	NA^e^	MCR-1	NC
2014	Russia	*Escherichia coli*	Peritoneal fluid	>4	0.12	2	0.03	0.25	ST156	MCR-1	TEM-OSBL; CTX-M-1
2014	South Africa	*Escherichia coli*	Wound	4	0.03	0.5	0.03	0.25	ST602	MCR-1	NC
2014	Spain	*Escherichia coli*	Peritoneal fluid	>4	0.12	0.25	0.015	0.5	ST117	MCR-1	NC
2014	Spain	*Escherichia coli*	Blood	4	1	64	0.12	2	ST167	MCR-1	TEM-OSBL
2014	Taiwan	*Escherichia coli*	Wound	4	0.25	32	0.06	0.25	ST117	MCR-1	TEM-OSBL; CTX-M-161; CMY-2
2014	Thailand	*Klebsiella pneumoniae*	Wound	4	0.5	64	0.06	0.5	NA	MCR-3.1	SHV-OSBL; CTX-M-55
2015	Argentina	*Escherichia coli*	Urine	4	0.12	0.5	0.03	0.25	ST48	MCR-1.5	NC
2015	Argentina	*Escherichia coli*	Peritoneal fluid	8	0.25	8	0.06	0.5	Novel^f^	MCR-1	CTX-M-2
2015	Colombia	*Escherichia coli*	Wound	4	0.12	0.25	0.03	0.5	ST744	MCR-1	NC
2015	Malaysia	*Escherichia coli*	Blood	4	0.03	0.25	0.03	0.5	ST2705	MCR-1	NC
2015	Malaysia	*Escherichia coli*	Wound	4	0.12	4	0.03	0.25	ST5907	MCR-1	TEM-OSBL; CTX-M-65
2015	Malaysia	*Escherichia coli*	Peritoneal fluid	4	0.06	0.12	0.03	0.12	ST7187	MCR-1	NC
2015	Spain	*Escherichia coli*	Endotracheal aspirate	4	0.12	0.25	0.03	1	ST88	MCR-1	NC
2015	Thailand	*Escherichia coli*	Wound	4	0.5	>128	0.12	2	ST1193	MCR-1	CMY-2
2015	Thailand	*Escherichia coli*	Blood	4	0.12	8	0.03	0.25	ST117	MCR-3.2	TEM-OSBL; CTX-M-55
2015	Thailand	*Escherichia coli*	Abscess	4	0.12	16	0.06	0.25	ST410	MCR-3.1	CTX-M-55
2015	Venezuela	*Escherichia coli*	Abscess	4	0.12	0.25	0.03	0.5	ST7973	MCR-1	NC
2016	Brazil	*Escherichia coli*	Peritoneal fluid	4	0.12	0.25	0.03	0.25	Novel^g^	MCR-1	NC
2016	Germany	*Escherichia coli*	Wound	4	0.12	0.25	0.03	0.25	ST1775	MCR-1	NC
2016	Poland	*Escherichia coli*	Wound	4	0.12	0.25	0.06	0.25	ST12	MCR-1	NC
2016	Thailand	*Escherichia coli*	Blood	4	0.12	16	0.12	0.12	ST4546	MCR-3.1	TEM-OSBL; CTX-M-55

^a^MICs performed via broth microdilution (13); CST, colistin; CAZ, ceftazidime; CAZ-AVI, ceftazidime with 4 μg/mL avibactam; MEM, meropenem; TGC, tigecycline.

^b^As part of INFORM, meropenem non-susceptible, ceftazidime-resistant, and phenotypically positive ESBL isolates were screened for genes encoding acquired extended-spectrum β-lactamases (ESBLs), AmpC β-lactamases, serine carbapenemases (KPC, OXA-48, GES), and metallo-β-lactamases (MBL) by PCR and DNA sequencing as previously described (16).

^c^NC = not characterized

^d^OSBL = original spectrum β-lactamase (eg. TEM-1, SHV-1, SHV-11)

^e^NA = not applicable

^f^Single-locus variant (novel *fumC*) of *E*. *coli* ST117

^g^Single-locus variant (novel *purA*) of pathogenic *E*. *coli* ST131

As part of the INFORM surveillance program, organisms non-susceptible to meropenem, resistant to ceftazidime, and/or positive for ESBL activity qualify for β-lactamase gene screening. Thirteen of the 29 *mcr* positive isolates qualified and were screened for genes encoding acquired ESBLs, AmpC β-lactamases, serine carbapenemases (*bla*_KPC_, *bla*_OXA-48_, *bla*_GES_), and metallo-β-lactamases by PCR and DNA sequencing, as previously described [16]. Nine *mcr*-positive isolates were found to carry CTX-M-type ESBLs either alone or in combination with AmpC-type β-lactamases and/or original-spectrum β-lactamases (OSBL) of the TEM or SHV type. Four possessed a CMY-2 AmpC-type enzyme either alone or with a TEM-OSBL, and in one case with a CTX-M-161 enzyme. None of the *mcr*-positive isolates carried carbapenemases. Of note, each of the four *mcr-*3 gene family-harboring isolates also carried the CTX-M-55 ESBL variant, known to be common in Asia especially in *E*. *coli* isolated from veterinary sources [17].

All *mcr* containing isolates were susceptible to meropenem (MIC < 2 μg/mL) and doripenem (MIC < 2 μg/mL), and 62.1% (18/29) were susceptible to both ceftazidime (MIC < 8 μg/mL) and aztreonam (MIC < 8 μg/mL) by CLSI breakpoints [18]. However, the addition of 4 μg/mL avibactam rendered 100% of the isolates susceptible (MIC < 8 μg/mL) to ceftazidime (using FDA recommended breakpoints [19]). All isolates harboring *mcr* were also susceptible (MIC ≤ 2 μg/mL) to tigecycline (using FDA recommended breakpoints [20]). The *in vitro* activity of several antimicrobials against the full set of 908 colistin-resistant isolates is given in Table 2. Ceftazidime-avibactam, along with tigecycline, were the most active agents against these isolates. The addition of avibactam to ceftazidime rendered 97.5% of the population susceptible (FDA breakpoints [19]), as compared to just 43.8% susceptibility with ceftazidime alone (CLSI breakpoints [18]).

**Table 2:** *In vitro* activity of selected antimicrobials against 908 colistin-resistant *Enterobacteriaceae* collected worldwide during 2014–2016.

Drug^a^	MIC Interpretive criteria (S/I/R) ^a^	% Susceptible	% Intermediate	% Resistant	MIC _50_ μg/mL	MIC _90_ μg/mL	MIC Range μg/mL
Amikacin	≤16/32/≥64	78.6	11.3	10.1	2	> 32	0.5 - >32
Ceftazidime	≤4 /8/≥16	43.9	2.0	54.1	32	> 128	≤0.015 - >128
Ceftazidime-avibactam^b^	≤8 /na/≥16	97.7	na	2.3	0.25	2	≤0.015 - >128
Colistin	≤2 /na/≥4	0	na	100.0	8	> 8	4 - >8
Levofloxacin	≤2 /4 /≥8	52.6	2.9	44.5	2	> 8	0.015 - >8
Meropenem	≤2 /4/≥8	70.4	3.2	26.5	0.12	> 8	0.008 - >8
Tigecycline	≤2 /4/≥8	95.6	4.0	0.4	0.5	2	0.03–8

^a^MICs were interpreted according to CLSI breakpoints [18], with the exception of ceftazidime-avibactam, for which MICs were interpreted using criteria according to the FDA [19], colistin for which EUCAST breakpoints were utilized [14] and tigecycline, for which MICs were interpreted using FDA criteria [20]; S, susceptible; I, intermediate; R, resistant; na, not applicable (no intermediate breakpoint).

^b^Avibactam concentration fixed at 4 μg/mL

The *mcr*-positive *E*. *coli* were distributed among several lineages, with the ST10 clonal complex (including ST167, ST744 and ST48) the most abundant (n = 6). *mcr*-harboring *E*. *coli* from this group has been reported on numerous occasions, for example ST10 from human clinical samples in China [21], ST744 from human and cattle-associated samples in Europe [22, 23], ST167 from human infections in Spain and China [24, 25], as well as ST48 from hospital sewage and human clinical samples, in China and Switzerland, respectively [26, 27]. Additional worldwide clones previously shown to harbor *mcr* were also confirmed here, and include ST641 [28], ST410 [29,30], and ST156 [31, 32]. Our screening identified two *mcr*-harboring ST117 *E*. *coli* (and a ST117 single-locus variant with a novel *fumC*), one of which carried the MCR-3.2 gene. ST117 is a clonal group associated with poultry disease [33] and *mcr-*type genes have only rarely been observed in this clone [27, 34]. Of particular interest, one isolate from Brazil typed as a single locus variant (novel *purA*) of the pathogenic *E*. *coli* ST131 [35]. ST131 often exhibits an extended spectrum β-lactamase (ESBL) phenotype and frequently possess CTX-M-15; however, this Brazilian isolate was susceptible to third-generation cephalosporins. In general, the fact that *mcr*-type genes have been found in *E*. *coli* of such diverse STs from food, human and animal specimens suggests the spread of these genes is linked more to successful plasmids and mobile elements rather than single specific *E*. *coli* clones [27].

Overall, the prevalence of *mcr* observed here is in accordance with previous reports from large global surveillance studies. For example, Castanheira, et al. noted that 4.9% (19/390) of a worldwide colistin-resistant collection of *E*. *coli* and *K*. *pneumoniae* from the SENTRY program contained *mcr-*1, and 32.3% (19/59) of the resistant *E*. *coli* contained this gene [36]. *mcr* was also enriched in the colistin-resistant *E*. *coli* population examined here, as 42.2% (27/64) of the resistant isolates from this species harbored *mcr* with the remainder presumably possessing a chromosomally-encoded resistance determinant. It should be noted that *mcr* has been discovered in isolates susceptible to colistin [37], so the actual frequency of occurrence could be higher. In this study, *mcr*-1 was observed exclusively in *E*. *coli* except for an *E*. *cloacae* isolate originating from Portugal. Until recently, *mcr*-1 positive *E*. *cloacae* were only reported from Asia [38, 39]; however, the geographic range was expanded with the discovery of a clinical *E*. *cloacae* isolate with *mcr*-1 in France [40]. The *mcr*-3 harboring *E*. *coli* and *K*. *pneumoniae* from Thailand confirm the previous report of the presence of this gene in clinical isolates from this country [9]. Finally, finding *mcr-5* in a Colombian *E*. *coli* clinical isolate expands both its geographic and host range, as at the time of this writing *mcr*-5 has only been confirmed in *Salmonella enterica* Paratyphi B isolated from food animals and food products in Germany, and in *E*. *coli* from porcine clinical specimens in Japan [41]. This gene was found *in silico* to be present the genome of a *Cupriavidus gilardii* from the U.S., and *mcr*-5 has been reported to be located on a unique Tn3-type transposon in both *S*. *enterica* Paratyphi B and *C*. *gilardii* [11]. Although we did not sequence this complete region, the forward *mcr*-5 flanking primer utilized to amplify the full coding region overlaps the 3’ end of the chromate reductase gene, *chr*B, directly upstream of *mcr*-5 in the Tn3-type transposon, and the reverse flanking primer anneals to the 5’ portion of the MFS-type transporter gene, immediately downstream of *mcr*-5 in the transposon arrangement [11], suggesting a similar genetic orientation in this Colombian strain.

In summary, this report confirms the global spread of *mcr*. Notably we did not find the co-existence of *mcr* with any carbapenemase genes, although co-carriage is being increasingly reported, including *mcr*-1 with *bla*_NDM_ in *Enterobacteriaceae* from the U.S. and China [32, 42–46], as well as *mcr-*1 and *bla*_KPC_ in isolates from Singapore [47]. Continual surveillance of this recently recognized threat to public health is warranted as MDR bacteria that acquire *mcr* will leave few treatment options.
